# An Integrated Model Based on Gut Microbiota and APOE Genotype for Predicting Dementia Risk

**DOI:** 10.3390/brainsci16080834

**Published:** 2026-08-06

**Authors:** Sehee Lee, Sun Hwa Hong, You Jin Nam, Yong Hyuk Cho, Sang Joon Son, Chang Hyung Hong

**Affiliations:** Department of Psychiatry, Ajou University School of Medicine, Suwon 16499, Republic of Korea; shlee1@ajou.ac.kr (S.L.); sh9@ajou.ac.kr (S.H.H.); yjnam@ajou.ac.kr (Y.J.N.); brainist.cho@gmail.com (Y.H.C.); sjsonpsy@ajou.ac.kr (S.J.S.)

**Keywords:** gut microbiota, dementia, APOE, cross-sectional classification, integrated model

## Abstract

**Highlights:**

**What are the main findings?**

- Dementia cases showed a higher frequency of APOE ε4 carriage, and participants classified as having moderate-to-high genetic risk had altered gut microbial patterns, including greater relative abundance of Streptococcus, Akkermansia, and Fusicatenibacter.
- The model combining clinical, pathological, genetic, and microbiome variables showed a numerically higher cross-validated AUC than models constructed from fewer data domains; this finding reflects cross-sectional dementia classification rather than prediction of future incident dementia.

**What are the implications of the main findings?**

- The findings support further evaluation of integrated, multi-domain approaches for distinguishing current dementia status, while prospective longitudinal validation is required before the framework can be used for future-risk prediction or clinical decision-making.
- The genotype-associated microbial differences observed in this cohort should be regarded as exploratory associations. Their relevance to personalized prevention or microbiome-directed intervention remains to be established in prospective studies.

**Abstract:**

Background: Dementia develops through the combined influence of genetic vulnerability, biological processes, and environmental exposures. The apolipoprotein E (APOE) ε4 allele is a well-known genetic contributor to dementia risk, and growing evidence links gut microbial alterations to cognitive decline and cerebrovascular-related pathology. Nevertheless, studies jointly evaluating genetic, microbiome, and clinical information remain relatively scarce. This study examined an integrated framework combining APOE genotype and gut microbiome data for cross-sectional dementia classification. Methods: We analyzed 292 participants representing three cognitive stages: subjective memory impairment (SMI), mild cognitive impairment, and dementia. Clinical variables, APOE genotype, and gut microbial metagenomic profiles were examined. Associations among genetic risk, Alzheimer’s disease pathology, and brain structural changes were assessed, and multivariable models were used to distinguish participants with dementia from those with SMI or MCI. Results: APOE ε4 carriage was most frequent among participants with dementia, while no ε4 carriers were observed in the SMI group. Gut microbial profiles differed according to the dementia-related genetic-risk category (mild vs. moderate-to-high). The fully integrated model showed a numerically higher cross-validated AUC than models constructed from fewer data domains. Streptococcus, Akkermansia, and Fusicatenibacter were more abundant in the moderate-to-high genetic-risk group; these taxon-level findings were exploratory and based on nominal *p*-values. Conclusions: The findings support an exploratory integrated framework for cross-sectional dementia classification based on genetic and gut microbiome information. Independent longitudinal and multicenter validation is required before the framework can be interpreted as predicting future dementia risk or supporting personalized clinical decisions.

## 1. Introduction

Dementia represents a growing public health issue worldwide, particularly in aging societies. Beyond its clinical consequences for affected individuals, it places considerable psychological, medical, and economic burdens on families and healthcare systems. Although advances have been made in neuroimaging, molecular biomarkers, and disease-modifying treatments, recognizing high-risk individuals remains difficult during preclinical or prodromal phases such as subjective memory impairment (SMI) and mild cognitive impairment (MCI) [1,2]. Patients who report subtle cognitive symptoms follow diverse clinical trajectories: some progress to dementia, whereas others remain stable or recover. This heterogeneity indicates the need for risk-stratification models that reflect the multifactorial mechanisms underlying cognitive decline [3,4].

Current evidence supports the view that dementia is not driven by a single pathway but rather by interactions among genetic predisposition, neurodegenerative changes, cerebrovascular damage, metabolic disturbances, immune-related alterations, and environmental exposures [5]. Because Alzheimer’s disease (AD) is the leading cause of dementia, genetic determinants of AD also contribute to dementia vulnerability. Among these, the apolipoprotein E (APOE) ε4 allele is the most established common genetic risk factor and is strongly linked to increased AD and dementia risk [6]. APOE is involved in lipid transport, synaptic function, neuroinflammation, and amyloid-β processing, and the risk of AD rises as the number of ε4 alleles increases. However, APOE ε4 alone does not determine disease onset: Many carriers remain free of dementia, while many patients with dementia do not carry ε4 [7]. These observations suggest that APOE genotype has limited stand-alone predictive value, especially when dementia emerges from multiple interacting biological processes.

The gut microbiota has recently garnered attention as a modifiable biological system related to cognitive impairment and dementia. Dysbiosis, or disruption of gut microbial composition and function, has been associated with inflammation, metabolic imbalance, and neuroimmune activation [8,9]. Through the gut–brain axis, the intestinal environment communicates bidirectionally with the central nervous system via neural, endocrine, and immune pathways. Microbial communities may affect brain function by regulating short-chain fatty acids (SCFAs), tryptophan metabolism, and neuroactive molecules such as gamma-aminobutyric acid and serotonin [10,11]. Previous experimental and clinical studies indicate that AD is accompanied by gut microbial alterations, including reductions in SCFA-producing beneficial bacteria and increases in taxa linked to inflammation. These microbiome changes may interact with host genetic background, including APOE genotype, and influence protein aggregation, microglial activation, and blood–brain barrier integrity [12].

Although APOE genotype and gut microbiota have each been studied in relation to dementia, relatively few studies have evaluated them jointly within an integrated classification framework. Genomic–microbiome multi-omics approaches remain limited in this field [9,10,11]. Examining APOE-related genetic variation together with microbiome-derived community differences may provide complementary information beyond models based on a single domain [9,12]. Accordingly, this study sought to identify gut microbial features associated with dementia-related genetic risk and to determine whether incorporating these features with APOE genotype and clinical variables improves cross-sectional dementia classification. Because the study was not longitudinal, it was not designed to predict incident dementia or establish the clinical utility of personalized prevention strategies.

## 2. Materials and Methods

### 2.1. Participants

#### 2.1.1. Study Design and Ethical Approval

The present analysis used data from the Biobank Innovations for Chronic Cerebrovascular Disease with Alzheimer’s Disease Study (BICWALZS), a national biobank initiative supported by the Korea Disease Control and Prevention Agency. BICWALZS was initiated in 2016 through collaboration between Ajou University School of Medicine and the National Biobank of Korea to collect and utilize human biospecimens together with real-world clinical information from individuals with SMI, MCI, AD dementia, and subcortical vascular dementia. The Ajou University Hospital Institutional Review Board approved the study protocol (Suwon, Republic of Korea; approval number AJOUIRB-SUR-2021-038). All participants and, when applicable, their legal guardians provided written informed consent before participation. The BICWALZS cohort is registered in the Korean National Clinical Trial Registry CRIS (KCT0003391). The original study plan was approved by the Institutional Review Board before study initiation (AJIRB-BMR-SUR-16-362), and all procedures complied with International Council for Harmonisation Good Clinical Practice guidelines [13].

#### 2.1.2. Recruitment and Baseline Assessments

The study included 292 participants enrolled through the Memory Clinic of Ajou University Hospital and the Suwon Mental Health Center for the Elderly in Gyeonggi-do, Republic of Korea. All individuals were Koreans of East Asian ancestry. Fecal specimens were obtained and stored for subsequent microbiome profiling. To support diagnostic classification, participants underwent structural brain MRI and amyloid PET imaging. Baseline clinical information and blood samples were additionally collected for cognitive and biochemical evaluation [13].

#### 2.1.3. Clinical Diagnosis and Neuroimaging-Based Classification

Clinical diagnosis was assigned using established international criteria. SMI was defined by self- and/or informant-reported cognitive decline in the absence of objective impairment on standardized cognitive testing or functional decline in daily activities. MCI was diagnosed according to the expanded Mayo Clinic criteria. AD was classified using the 2011 National Institute on Aging-Alzheimer’s Association (NIA-AA) criteria, whereas vascular dementia was identified using the Diagnostic and Statistical Manual of Mental Disorders, Fifth Edition (DSM-5) criteria for major vascular neurocognitive disorder.

Cognitive impairment was determined using z-scores adjusted for age, sex, and education, with impairment defined as a z-score of −1 or lower. Participants with amnestic MCI or AD were assigned to the AD-related cognitive impairment group, while individuals with severe white matter hyperintensities or other major vascular lesions were not included in this category. Subcortical vascular MCI and subcortical vascular dementia were grouped as vascular cognitive impairment. Participants with cortical amyloid positivity on PET, or with infarctions or white matter hyperintensities attributable to radiation injury, multiple sclerosis, vasculitis, or leukodystrophy, were excluded from the vascular cognitive impairment group [13].

### 2.2. Gut Microbiota Analysis

#### 2.2.1. Bacterial DNA Extraction

For bacterial DNA extraction, fecal material was suspended in 6 mL of phosphate-buffered saline and incubated for 24 h before filtration through a cell strainer. The filtrate was centrifuged at 10,000× *g* for 10 min at 4 °C, and the resulting bacterial pellet was isolated. The pellet was then resuspended in 200 µL of phosphate-buffered saline. The supernatant was passed through a 0.2 µm syringe filter and heated at 100 °C for 40 min in a heat block. Remaining particulate matter was removed by centrifugation at 18,312× *g* for 30 min at 4 °C. DNA was purified from the bacterial fraction using the DNeasy PowerSoil Pro Kit (Qiagen, Hilden, Germany) according to the manufacturer’s protocol. The purified DNA was used for library construction, and sample-specific barcode sequences were assigned to the 16S rDNA amplicons.

#### 2.2.2. Analysis of Microbial Community Composition

Sequencing was conducted on the Illumina MiSeq platform. Paired-end reads were first processed with Cutadapt (v1.1.6) to remove adapter sequences [14]. Filtered reads were merged using CASPER, and sequences that met the predefined Phred quality-score criteria were retained for downstream analysis [15,16]. Taxonomic assignment was performed by aligning processed sequences to the SILVA database (v138). Merged reads outside the 350–550 bp length range were discarded. Chimeras were detected using VSEARCH (v2.0.3) with a reference-based approach against the SILVA (v138) gold database [17,18].

### 2.3. Data Preprocessing and Diversity Analysis

#### 2.3.1. Alpha and Beta Diversity Analysis

Microbial diversity was assessed at both within-sample and between-sample levels. Alpha diversity was summarized using Observed OTUs, Chao1 richness, Shannon diversity, and Simpson dominance indices. Beta diversity was examined by principal coordinate analysis (PCoA) based on Bray–Curtis dissimilarity to visualize sample relationships and clustering across taxonomic ranks from phylum to species. Group differences were tested using Student’s *t*-test or the Wilcoxon rank-sum test for two-group comparisons, and ANOVA or the Kruskal–Wallis test for comparisons involving three or more groups. Taxon-level comparisons were exploratory. Taxa displayed in the main figure were selected using a nominal *p*-value threshold of <0.05 and a relative-abundance threshold of >1% in at least one group. No formal false-discovery-rate correction was applied; therefore, these findings should be interpreted cautiously and require independent confirmation.

#### 2.3.2. Data Preprocessing

Statistical analyses were performed using R (v4.3.3) and Python (v3.12). Variables were coded as follows: sex (0 = female, 1 = male), diabetes (0/1), Alzheimer’s pathology (1 = negative, 2 = positive), dementia-related brain changes (1 = mild, 2 = moderate or greater), dementia-related genetic risk (1 = mild, 2 = moderate or greater), cognitive impairment severity (1 = SMI, 2 = MCI, 3 = dementia), Clinical Dementia Rating (CDR; 1 = mild, 2 = moderate or greater), and ADL status (1 = no problem, 2 = problem). Age was treated as a numeric variable and mean-centered. APOE ε4 carrier status was defined from genotype information, with E2/E4, E3/E4, and E4/E4 classified as carriers and all other genotypes classified as non-carriers. Participants were classified into mild and moderate-to-high genetic-risk groups based on APOE ε2 carrier status. APOE ε2 carriers, including individuals with the ε2/ε3 or ε2/ε4 genotype, were categorized as the mild-risk group (*n* = 40), whereas APOE ε2 non-carriers with the ε3/ε3, ε3/ε4, or ε4/ε4 genotype were categorized as the moderate-to-high-risk group (*n* = 252). This ε2 carrier-based classification was distinct from APOE ε4 carrier status, which was analyzed separately. Complete-case analysis was performed separately for each model. Model A included 292 participants, Model B included 291, and Model C included 289; thus, one and three participants, respectively, were excluded because at least one model-specific variable was missing. Multicollinearity was evaluated using the variance inflation factor (VIF), and variables with VIF values above 10 were excluded from multivariable models. Because only one participant was excluded from Model B and three from Model C, the missing-data mechanism could not be formally assessed; therefore, missingness cannot be assumed to have occurred at random.

#### 2.3.3. Integrated Risk Factor Analysis and Predictive Modeling of Dementia

Dementia status was defined as cognitive impairment severity level 3 versus levels 1–2 and modeled using multivariable logistic regression. Accordingly, the outcome represented cross-sectional dementia classification rather than prediction of future incident dementia. The models included age, sex, diabetes, education, BMI, Alzheimer’s pathology, brain structural changes, genetic risk, APOE ε4 carrier status, and selected microbiome genera according to the model specification. Adjusted odds ratios and 95% confidence intervals were estimated. Model performance was evaluated by calibration using the Hosmer–Lemeshow test and by discrimination using the area under the receiver operating characteristic curve (AUC), calculated from ten repeated five-fold cross-validations. To visualize the distribution and overlap of key risk domains across SMI, MCI, and dementia stages, stacked proportion plots were generated for Alzheimer’s pathology, brain structural changes, and genetic risk. Group differences were examined using chi-square tests and, when appropriate, Cochran–Armitage or Jonckheere–Terpstra trend tests; effect sizes were summarized with risk differences and Cramer’s V. Sensitivity analyses used CDR status (moderate or greater vs. mild) as an alternative outcome. These procedures provided internal performance estimates only. No independent external validation dataset was available, and repeated cross-validation cannot fully eliminate optimism arising from model development and evaluation within the same cohort.

The microbiome genera entered into the fully integrated model were selected from the exploratory taxon-level comparison using the prespecified reporting criteria of nominal *p* < 0.05 and relative abundance >1% in at least one genetic-risk group. Because taxon selection and model evaluation were conducted in the same cohort, the estimated performance may be optimistic. Formal pairwise comparison of cross-validated ROC curves, confidence intervals for the mean cross-validated AUC, calibration plots, and decision-curve analysis were not available in the present analysis; consequently, differences between model AUCs are described as numerical rather than statistically confirmed improvements. Specifically, selecting microbiome taxa using the full dataset before cross-validation may have introduced information leakage and feature-selection bias. Future studies should perform taxon selection within each training fold of a nested cross-validation framework or use an independent training dataset.

## 3. Results

### 3.1. Demographic and Clinical Characteristics of Study Participants

A total of 292 participants were included in the study and classified into three cognitive-stage groups: SMI (*n* = 19), MCI (*n* = 203), and dementia (*n* = 70) (Table 1). As shown in Table 1, the cognitive stage classification in the study cohort showed larger between-group differences in AD-related pathology and biomarkers than in demographic factors. Specifically, individuals in the dementia group showed a markedly higher prevalence of Alzheimer’s pathology and APOE ε4 carriage, accompanied by substantially worse cognitive and functional performance. These clinical features paralleled a consistent biomarker signature characterized by greater amyloid PET positivity and amyloid load, more severe medial temporal lobe atrophy, and elevated blood-based markers of tau pathology, neuroaxonal injury, and astroglial activation. These between-group patterns were more pronounced for AD pathology and biomarker measures than for demographic variables.

Multivariable logistic regression analysis further demonstrated that Alzheimer’s pathology positivity and dementia-related brain structural changes were the strongest independent predictors of dementia status, even after accounting for other clinical covariates (Table 2).

### 3.2. Stage-Wise Distribution Patterns of Major Dementia-Related Risk Factors and Overlapping of Major Risk Factors

**Figure 1:**
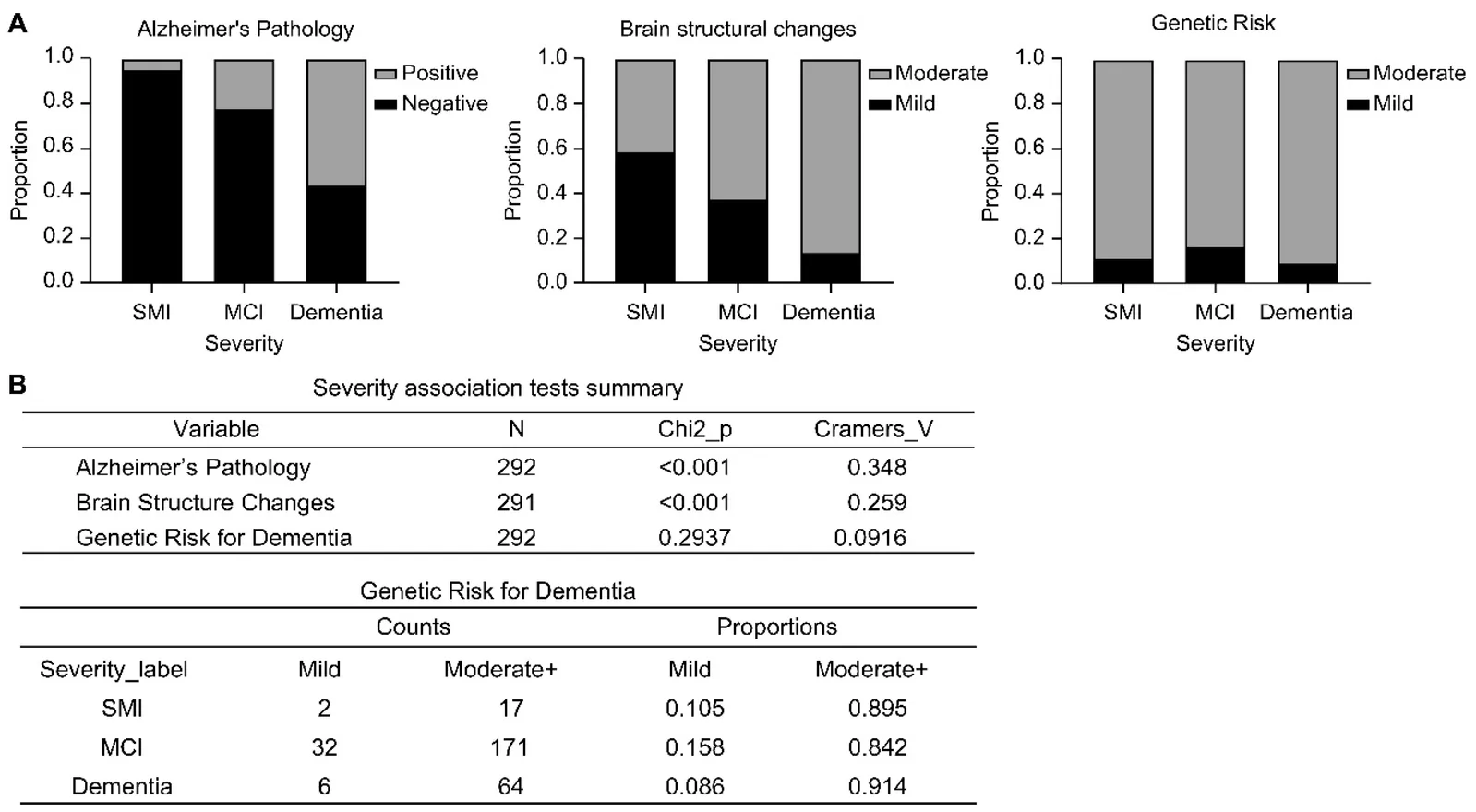
Distribution of major risk factors across cognitive impairment stages. (**A**) Stacked bar plots showing the stage-wise distribution of three major dementia-related risk domains across cognitive stages (SMI, MCI, and Dementia): Alzheimer’s pathology (positive vs. negative), dementia-related brain structural changes (moderate-to-high vs. mild), and genetic risk for dementia (moderate-to-high vs. mild). The *x*-axis represents the cognitive severity group, and the *y*-axis represents the proportion of participants. Bars represent proportions within each cognitive stage. (**B**) Summary of association tests between cognitive stage and each risk domain using chi-square tests, with effect sizes reported as Cramer’s V (N indicates the number of participants with non-missing data for each comparison). Counts and proportions for each stage.

We first assessed how the major dementia-related risk domains were distributed across cognitive stages by comparing Alzheimer’s pathology, brain structural changes, and genetic risk among participants with SMI, MCI, and dementia (Figure 1). Alzheimer’s pathology positivity increased clearly with advancing cognitive stage, supporting its close relationship with cognitive impairment severity. A similar pattern was observed for moderate-to-severe brain structural changes, which became more common as cognitive severity progressed, suggesting progressive accumulation of brain vulnerability along the clinical continuum (both chi-square tests *p* < 0.001). These findings indicate that key risk domains tend to concentrate in the dementia group and increasingly overlap as cognitive impairment worsens. By contrast, the distribution of mild versus moderate genetic risk did not differ significantly across stages (chi-square *p* = 0.294), suggesting that cognitive decline in this cohort was more strongly linked to AD pathology and structural brain changes than to a graded shift in genetic risk alone.

**Figure 2:**
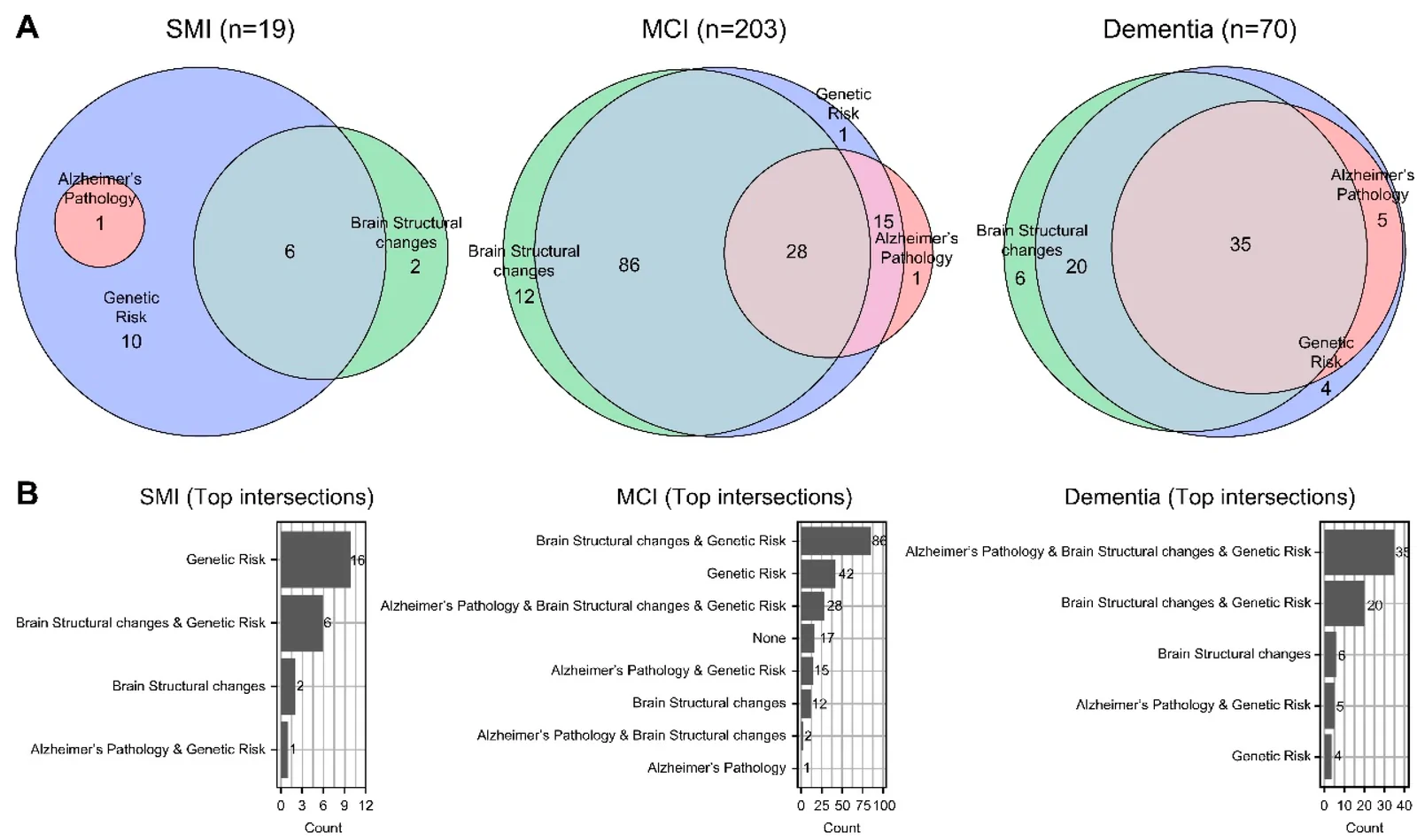
Stepwise overlap of major dementia related risk domains. (**A**) Venn diagrams depicting within-stage overlap among three major dementia-related risk domains stratified by cognitive stage (SMI, MCI, and dementia). Numbers indicate the count of participants within each domain combination. (**B**) Horizontal bar plots showing the most frequent intersection patterns (top combinations) within each cognitive stage, with counts displayed at the end of each bar. Risk domains were defined as follows: Alzheimer’s pathology positive (binary), brain structural changes classified as moderate-to-severe, and genetic risk categorized as moderate-to-high.

The overlap among Alzheimer’s pathology, brain structural changes, and genetic risk factors within each group is illustrated using a Venn diagram (Figure 2). In the SMI group, genetic risk was frequently observed in isolation, whereas the dementia group showed a greater proportion of cases in which all risk factors were present concurrently (central overlap). The most frequent intersection patterns within each stage are further summarized in the corresponding bar plots (Figure 2B). Together, these results indicate that as cognitive impairment progresses, major risk domains tend to converge rather than appear as a single isolated domain.

### 3.3. Distinct Gut Microbiota Signatures in Genetically High-Risk Individuals

**Figure 3:**
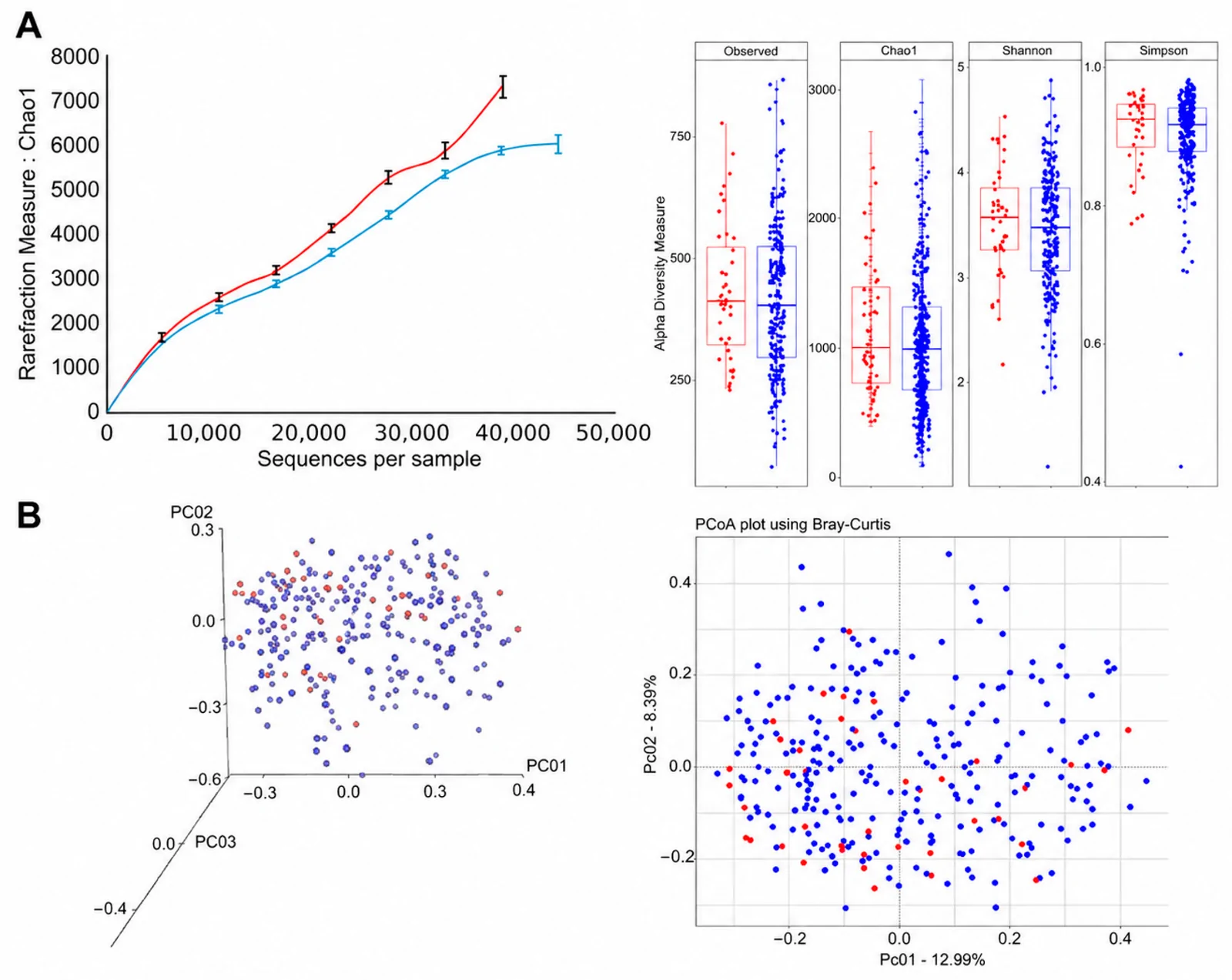
Gut microbial diversity according to genetic-risk level. (**A**) Alpha-diversity profiles, including rarefied Chao1, Observed OTUs, Chao1, Shannon, and Simpson indices. (**B**) Principal coordinate analyses based on Bray–Curtis dissimilarity. Red indicates the mild-risk group and blue indicates the moderate-to-high-risk group.

Alpha diversity analysis revealed the internal richness and complexity of the gut microbiota. Detailed comparisons of observed OTUs, Chao1, Shannon, and Simpson indices were performed to identify differences in microbial structure between risk groups. All diversity indices were significantly higher in the mild-risk group (*p* < 0.05, Wilcoxon rank-sum test). Beta diversity analysis, visualized using 3D and 2D PCoA plots based on Bray–Curtis dissimilarity, revealed the spatial distribution and clustering of microbial communities (Figure 3).

**Figure 4:**
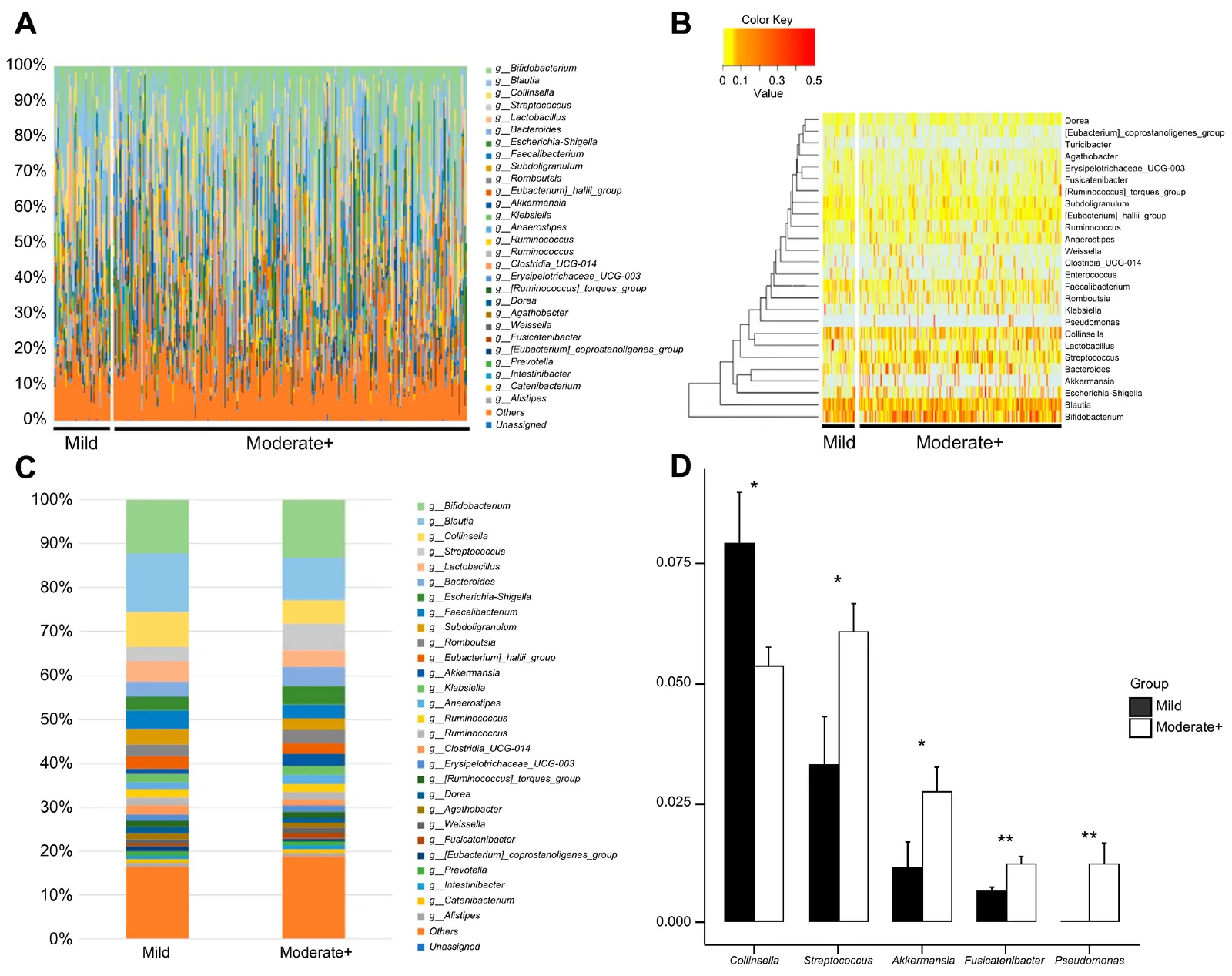
Genus-level gut microbiota profiles according to genetic-risk level. (**A**) Individual-level relative-abundance profiles. (**B**) Heatmap of genera meeting the reporting threshold. (**C**) Group-averaged composition. (**D**) Genera meeting the exploratory criteria of nominal *p* < 0.05 and >1% relative abundance in at least one group. * *p* < 0.05; ** *p* < 0.01. These findings were not adjusted for multiple comparisons and should be interpreted as exploratory.

To investigate microbiome differences associated with dementia-related genetic risk, we compared community profiles across multiple taxonomic levels. At the genus level, the moderate-to-high risk group showed higher relative abundances of Streptococcus, Akkermansia, Fusicatenibacter, and Pseudomonas, whereas Collinsella was relatively reduced compared with the mild-risk group. These taxa met the exploratory reporting criteria of nominal *p* < 0.05 and relative abundance >1% in at least one group (Figure 4D). Because no formal correction for multiple taxonomic comparisons was applied, these findings are presented as exploratory associations rather than validated microbial biomarkers. Results from the other taxonomic levels are summarized in Figure 3 and Figure 4.

### 3.4. Predictive Performance of Genotype–Microbiome Models for Dementia Classification

To evaluate cross-sectional dementia classification, we constructed a series of multivariable logistic regression models. All models included baseline clinical covariates (age, sex, diabetes, education level, and BMI), and additional variables were added sequentially. Model discrimination was summarized using the mean AUC and standard deviation from ten repetitions of five-fold cross-validation, and calibration was evaluated using the Hosmer–Lemeshow goodness-of-fit test. These estimates reflect internal cross-validation and do not represent external validation or prediction of incident dementia.

**Figure 5:**
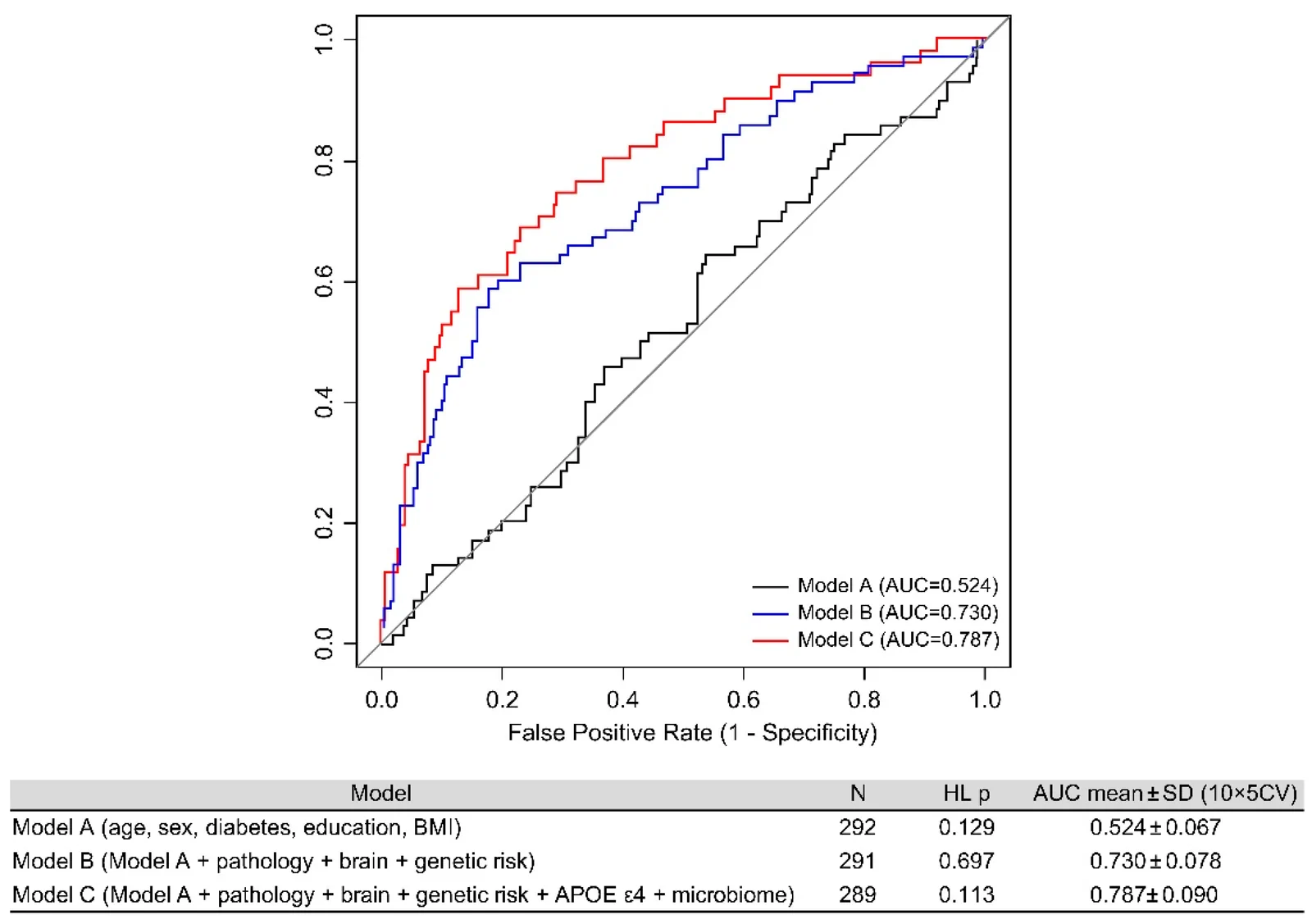
Predictive performance of genotype-microbiome models for dementia classification. Receiver operating characteristic curves comparing three multivariable logistic regression models for dementia classification. Discrimination was evaluated using the mean area under the ROC curve (AUC) with standard deviation obtained from ten repetitions of five-fold cross-validation, and calibration was assessed using the Hosmer–Lemeshow [19] test. The accompanying table summarizes sample size (N), HL *p*-values, and cross-validated AUC (mean ± SD) for each model.

The baseline clinical model (Model A) showed a cross-validated AUC of 0.524 ± 0.067 (HL *p* = 0.129). Model B, which additionally included Alzheimer’s pathology, dementia-related brain structural changes, and genetic risk, showed an AUC of 0.730 ± 0.078 (HL *p* = 0.697). Model C, which further included APOE ε4 carrier status and selected microbiome genera, showed the numerically highest AUC of 0.787 ± 0.090 (HL *p* = 0.113). Because formal pairwise tests of the cross-validated AUCs were not performed, these differences should not be interpreted as statistically established superiority. ROC curves for Models A–C are shown in Figure 5.

## 4. Discussion

In this cross-sectional cohort spanning SMI, MCI, and dementia, Alzheimer’s pathology and dementia-related brain structural changes showed the clearest stage-wise patterns. Genetic-risk categories did not differ significantly across cognitive stages, although microbiome composition varied between the mild and moderate-to-high genetic-risk groups. In the classification analyses, Model C showed the numerically highest internally cross-validated AUC. These results indicate complementary information across data domains, but they do not demonstrate prediction of future dementia or external generalizability.

The moderate-to-high genetic-risk group showed lower alpha diversity and differences in selected genera. Similar microbial patterns have been reported in cognitive decline and neurodegenerative disease [19,20,21,22]. However, the present data do not establish whether these differences preceded dementia, resulted from disease-related changes, or reflected differences in diet, medication, bowel habits, functional status, or other unmeasured factors.

Streptococcus, Akkermansia, and Fusicatenibacter were more abundant in the moderate-to-high genetic-risk group. Prior literature has linked these taxa to inflammatory, mucin-degrading, or short-chain-fatty-acid-related pathways [8,23,24,25,26]. These mechanistic interpretations derive from previous studies and cannot be inferred from the present cross-sectional associations. Furthermore, because the taxon-level analyses used nominal *p*-values without formal multiple-testing correction, the identified genera should be considered exploratory candidates requiring independent replication. Accordingly, the taxon-level findings and their biological interpretations should be regarded as hypothesis-generating associations that require independent validation.

The integrated model showed a numerically higher cross-validated AUC than the models containing fewer data domains. However, the improvement was not formally tested using paired comparisons of out-of-fold predictions, and AUC with the Hosmer–Lemeshow test alone does not establish clinical utility. Sensitivity, specificity, predictive values, calibration plots, decision-curve analysis, and external validation are needed before clinical application can be considered. In addition, the binary outcome was imbalanced, with 70 participants with dementia and 222 without dementia. Future studies should therefore report threshold-dependent performance measures, precision–recall metrics, and balanced accuracy in addition to ROC-AUC.

Taken together, the findings support further study of integrated genetic and microbiome information for cross-sectional dementia classification. They do not establish prospective risk prediction, personalized prevention, or a microbiome-directed intervention strategy. Such applications require prospective validation with prespecified models and clinically relevant thresholds.

Several limitations should be considered. First, the cross-sectional design precludes temporal or causal inference and does not permit prediction of incident dementia. Second, diet, antibiotics, probiotics, proton-pump inhibitors, metformin, statins, constipation, bowel habits, physical activity, nutritional status, and other relevant variables were not consistently available and were not incorporated into the models; residual confounding may therefore explain part of the observed microbiome differences. Third, the single-center cohort consisted exclusively of Korean participants, limiting external validity across other ethnic and clinical populations. Fourth, taxon-level comparisons used nominal *p*-values without formal multiple-testing correction, increasing the possibility of false-positive findings. Fifth, taxon selection and model evaluation were conducted within the same cohort, and repeated cross-validation cannot eliminate optimism bias. Sixth, complete-case analysis excluded one participant from Model B and three from Model C; although the amount of missingness was small, selection bias cannot be excluded. Seventh, the SMI group was substantially smaller than the MCI and dementia groups, reducing the precision of stage-specific comparisons. Finally, formal ROC comparisons, AUC confidence intervals, calibration plots, sensitivity, specificity, predictive values, and decision-curve analysis were not available; therefore, the model’s clinical utility and comparative superiority remain uncertain. Eighth, the genetic-risk grouping was based solely on APOE ε2 carrier status and was specific to the source dataset. Notably, individuals with the ε2/ε4 genotype were classified in the mild-risk group because they carried an ε2 allele, despite also carrying ε4. Therefore, this grouping should not be interpreted as a conventional or externally validated APOE risk hierarchy.

## 5. Conclusions

In summary, Alzheimer’s pathology and dementia-related structural brain changes were more closely associated with cognitive stage than general demographic or clinical characteristics. Participants with higher genetic risk showed exploratory genus-level microbiome differences. The model integrating pathology, structural brain changes, genetic risk, APOE ε4 carrier status, and selected microbiome genera showed the numerically highest internally cross-validated AUC for distinguishing dementia from SMI or MCI. Because this was a cross-sectional, single-center study without external validation or formal comparison of model AUCs, the framework should not be interpreted as predicting future dementia or supporting personalized prevention. Independent longitudinal and multicenter studies with prespecified analyses, comprehensive confounder assessment, and clinical-utility evaluation are required.

## Figures and Tables

**Table 1:** Demographic and clinical characteristics of study participants (*n* = 292).

	All Participants (*n* = 292)	SMI (*n* = 19)	MCI (*n* = 203)	Dementia (*n* = 70)	*p*-Value
Variables					
Age, y	72.5 (6.9)	72.7 (5.9)	72.4 (6.6)	72.8 (7.9)	0.908
Sex, female	88 (30.1)	4 (21.1)	59 (29.1)	25 (35.7)	0.389
Education, y	7.5 (4.7)	7.3 (5.0)	7.7 (4.7)	7.1 (4.7)	0.652
Body mass index	23.9 (3.4)	24.0 (3.9)	24.1 (3.3)	23.2 (3.4)	0.147
Comorbidity					
Diabetes	65 (22.3)	5 (26.3)	42 (20.7)	18 (25.7)	0.621
Alzheimer pathology	87 (29.8)	1 (0.1)	46 (22.7)	40 (57.1)	<0.001
Vascular pathology	96 (32.9)	6 (31.6)	59 (29.1)	31 (44.3)	0.065
Brain change	197 (67.5)	8 (42.1)	128 (63.1)	61 (87.1)	<0.001
APOE genotype, ε4 carrier	77 (26.4)	1 (0.1)	46 (22.7)	30 (42.9)	<0.001
MMSE score	23.0 (4.6)	26.4 (3.3)	24.2 (3.7)	18.8 (4.6)	<0.001
Neuroimaging-based biomarkers					
Amyloid PET positivity	87 (29.8)	1 (5.3)	46 (22.7)	40 (57.1)	<0.001
Amyloid PET centiloid	31.0 (47.8)	4.3 (7.6)	23.5 (40.5)	60.8 (60.0)	<0.001
Blood-based biomarkers					
pTau217	0.414 (0.392)	0.219 (0.133)	0.348 (0.310)	0.649 (0.524)	<0.001
NfL	34.94 (27.79)	27.50 (11.95)	33.18 (25.31)	42.04 (35.65)	0.002
GFAP	172.3 (86.1)	150.1 (72.2)	163.8 (81.4)	202.6 (96.0)	<0.001

Values are presented as mean (SD) for continuous variables and *n* (%) for categorical variables.

**Table 2:** Multivariate logistic regression analysis for dementia status.

Variables	OR	Beta	S.E.	*p*-Value
Age	0.945	−0.056	0.025	0.022
Sex	1.051	0.05	0.337	0.882
Education	0.938	−0.064	0.036	0.08
BMI	0.954	−0.047	0.046	0.303
Diabetes	1.388	0.328	0.362	0.365
Alzheimer’s pathology positive	6.407	1.857	0.352	<0.001
Brain structural changes	4.362	1.473	0.436	<0.001
Genetic risk	1.275	0.243	0.518	0.639

Results are presented as odds ratios (ORs) with 95% confidence intervals (CIs) and corresponding regression coefficients (β) with standard errors.

## Data Availability

The datasets generated and analyzed during the current study are not publicly available owing to the inclusion of sensitive patient information but can be made available by the corresponding author on reasonable request.

## Abbreviations

The following abbreviations are used in this manuscript:

APOE	Apolipoprotein E
AD	Alzheimer’s Disease
AUC	Area under the receiver operating characteristic curve
SMI	Subjective Memory Impairment
MCI	Mild Cognitive Impairment
OTUs	Operational Taxonomic Units
PCoA	Principal coordinate analysis
